# Low dose endotoxin priming is accountable for coagulation abnormalities and organ damage observed in the Shwartzman reaction. A comparison between a single-dose endotoxemia model and a double-hit endotoxin-induced Shwartzman reaction

**DOI:** 10.1186/1477-9560-4-13

**Published:** 2006-08-24

**Authors:** Sjoukje H Slofstra, Hugo ten Cate, C Arnold Spek

**Affiliations:** 1 Center for Experimental and Molecular Medicine, Academic Medical Center, Amsterdam, The Netherlands; 2 Department of Internal Medicine, Academic Hospital and Cardiovascular Research Institute, Maastricht, The Netherlands

## Abstract

The clinical response of sepsis to a systemic inflammatory infection may be complicated by disseminated intravascular coagulation or DIC. In order to experimentally study the syndrome of DIC, we aimed for a severe sepsis model complicated by disseminated coagulation. Most -simplified- experimental models describing coagulation abnormalities as a consequence of sepsis are based on single dose endotoxemia. The so called-Shwartzman reaction contrarily, is elicited by a low dose endotoxin priming followed by an LPS challenge and is characterized by pathological manifestations that represent the syndrome of DIC. In order to investigate whether the Shwartzman reaction is superior to a single endotoxin challenge as a model for sepsis-induced DIC and to determine what the pathological effect is of an encounter of low endotoxin prior to an LPS challenge, we undertook the present study.

In this study we demonstrate that low-dose endotoxin priming prior to an LPS challenge in the Shwartzman reaction is accountable for micro-vascular thrombosis in lung and liver and subsequent (multi-) organ failure, not observed after a single-dose endotoxin challenge, which indicates that the Shwartzman reaction is well suited-model to study sepsis-induced DIC adversities. Remarkably, only minor differences in the innate immune response were established between the single-dose endotoxin challenge and the Shwartzman reaction.

## Background

Severe bacterial infections may pass into the bloodstream where endotoxin or lipopolysaccharide (LPS) present on the outer membranes of (gram-negative) bacteria can provoke a systemic inflammatory response syndrome. The term sepsis is used to identify the continuum of the clinical response to such a systemic infection and sepsis may progress to severe sepsis or septic shock when additional organ dysfunction and hypoperfusion develop [1]. The initial manifestation of the infection is overwhelming inflammation, known as the innate immune response.

Furthermore, severe sepsis is complicated by disseminated intravascular coagulation (DIC), a syndrome characterized by micro-vascular thrombosis and (multi-) organ failure consequently increasing mortality rate [2]. Although a contributory relation between intravascular deposition of fibrin, as a result of the systemic activation of coagulation, and the development of organ damage is not firmly established, anticoagulant strategies are believed to benefit the condition of DIC [2,3]. Thus far, the restoration of the anti-coagulant protein C pathway by the infusion of recombinant human activated protein C (APC) is the only treatment strategy proven to improve sepsis-related mortality [4]. Interestingly, sub-group analysis indicated that patients who were classified as having DIC, (according to the DIC scoring system of the ISTH [5]) had a relatively greater benefit of APC treatment than patients who did not have overt DIC [6] which underscores the importance of coagulation derangement in the pathogenesis of severe sepsis/DIC. Large-scale, multi-centre, randomized controlled trials aimed at the restoration of the antithrombin (ATIII) [7] and tissue factor pathway inhibitor (TFPI) [8,9] anticoagulant pathways however, did not demonstrate a significant reduction in mortality in patients with sepsis, suggesting that targeting the aggravated coagulation response is not sufficient and additional strategies should be considered.

In order to study the pathogenic characteristics during the sepsis-induced DIC phenomenon we aimed for a murine endotoxin-induced model, with consequential coagulation abnormalities resembling (human) DIC. Endotoxemia models by a single LPS infusion are often used as a representative for sepsis as they mimic the initial innate immune response [10-12]. Although coagulation abnormalities have been described in such models [10,11], the Shwartzman reaction might be a better model projecting severe sepsis complicated by DIC [13].

The Shwartzman reaction can be elicited in experimental (animal) models by two consecutive injections of endotoxin and is characterized by platelet aggregation, vascular occlusion, inhibition of fibrinolysis, neutrophil accumulation, endothelial injury and variable degrees of apoptosis and necrosis in the microvasculature [14-17]. In order to investigate whether the Shwartzman reaction is superior to a single endotoxin challenge as a model for sepsis-induced DIC and to determine what the pathological effect is of an encounter of low endotoxin prior to an LPS challenge, we undertook the present study.

## Materials and methods

### Animals

10 Week old, female C57Bl/6 mice were purchased from Charles River (Someren, the Netherlands). All mice were maintained at the animal care facility at the Academic Medical Center according to institutional guidelines. Animal procedures were carried out in compliance with the Institutional Standards for Humane Care and Use of Laboratory Animals. The Animal Care and Use Committee of the Academic Medical Centre (Amsterdam, the Netherlands) approved all experiments.

### Experimental design

Mice were subjected to two consecutive injections; a low dose endotoxin priming injection of 5 μg *Serratia marcescens *LPS (Sigma-Aldrich, St. Louis, MO)(in 40 μl) or sterile saline control (no-priming) was given in the foot (intradermally) and was followed 24 h later by an intravenous LPS challenge (300 μg *Serratia marcescens *LPS)(in 100 μl) [18]. Mice receiving two endotoxin injections will develop a Shwartzman reaction. No-priming controls represent a single-dose endotoxemia model.

Mice were sacrificed at baseline levels (t = -24 h), 24 hours after priming (t = 0 h), 2 hours after challenge (t = 2 h) and 6 hours after challenge (t = 6 h). Each group contained 8 mice.

### Cytokine chemokine assay

IL-6, IL1β, KC and TNFα were measured using the Bio-Rad Bioplex system following manufacturer's instructions. In short, dyed beads conjugated with monoclonal antibodies are allowed to react with sample and a secondary detection antibody. Bio-Plex array reader illuminates the dyed beads and the distinct fluorescent signature allows the identification of each bead. A green laser in the array reader simultaneously excites the fluorescent reporter tag bound to the detection antibody in the assay. The amount of green fluorescence is proportional to the amount of analyte captured in the immunoassay. Extrapolating to a standard curve allows quantification of each analyte in the sample.

### Histological examination

Tissue (lung, liver and kidney) sections were fixed in 10% buffered formalin and embedded in paraffin; 4 μm thick sections were stained with haemotoxylin and eosin. Number of thrombi in liver and lung and necrotic areas in liver were scored for ten microscopic fields at a magnification of 25 ×.

### Enzyme assay

Creatinine and liver enzymes (ASAT/ALAT) in plasma (1:5 dilution in 0.9% sterile saline) were determined with commercially available kits (Sigma) using a Hitachi analyzer (Boehringer Mannheim, Mannheim, Germany) according to manufacturer's instructions.

### Statistical analysis

Statistical analyses were conducted using GraphPad Prism version 3.00, Graphpad software (San Diego, CA). Data are expressed as means ± SE. Comparison between two groups was analyzed using two-tailed Mann-Whitney U tests.

## Results

### Effect of low endotoxin priming on the innate immune response

Intravenous LPS infusion primarily results in an inflammatory reaction that reflects the innate immune response. Therefore we analyzed the inflammatory markers IL-1β, KC (neutrophil chemotactic protein), IL-6, TNFα, and IL-10 in plasma of mice subjected to a single intravenous LPS challenge (no-priming) or a double hit Shwartzman reaction (+24 h low dose endotoxin priming).

**Figure 1:**
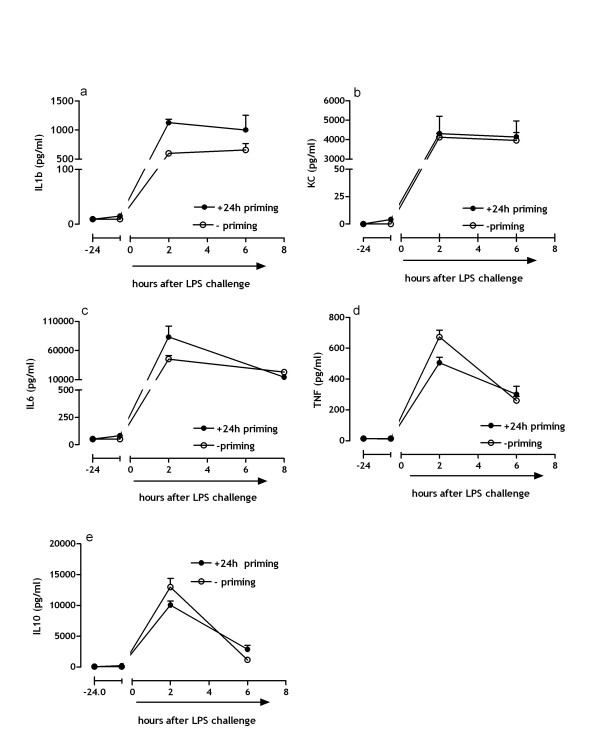
**Systemic innate immune response after LPS challenge**. (a) Represents the effect of low dose endotoxin priming on LPS induced IL-1β levels. (b) demonstrates KC levels, and panel (c) (d) and (e) represent respectively IL-6, IL-10 TNFα levels in plasma. (mean ± SE)

Two hours after LPS challenge IL-1β levels are notably increased in mice that were subjected to 24 hours of low dose endotoxin priming compared to the non-primed mice (p < .001) (fig 1a), the difference however, becomes less pronounced 6 hours after the LPS challenge (ns). Contrarily, KC and IL-6 levels are not evidently affected by 24 hours of low dose priming (fig 1b and 1c).

The inflammatory mediators, IL-6, TNFα and IL-10 are transiently increased in response to the LPS challenge. TNFα levels are significantly (p < .01) lower in mice subjected to low dose endotoxin priming compared to the non-primed mice 2 hours after LPS challenge (fig 1d); this effect nevertheless disappears in time. The level of the anti-inflammatory cytokine IL-10 is slightly but significantly (p < .05) lower in the non-primed mice compared to the mice who did receive the priming injection 6 hours after LPS challenge (fig 1e). These results indicate that 24 hours of low dose endotoxin priming has minor effects on the innate immune response towards an LPS challenge.

### Low dose endotoxin priming results in micro-vascular thrombosis

**Figure 2:**
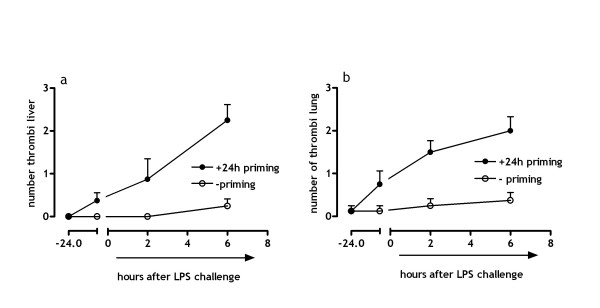
**Thrombi formation in liver and lung**. Formation of (micro-) thrombi after LPS challenge with and without 24 hours of low dose endotoxin priming. (a) Represents the number of thrombi in liver. (b) Demonstrates the number of thrombi in 10 microscopic fields (25×) in lung tissue. (mean ± SE)

The syndrome of DIC is characterized by coagulation abnormalities featured by micro-vascular thrombosis in target organs. The Shwartzman reaction is proposed to represent an endotoxin-induced model of DIC. Therefore, we determined the effect of low dose endotoxin priming on the formation of micro-vascular thrombi. Figure 2 demonstrates that LPS challenge induces a significant increase in the number of thrombi formed in the lung (fig 2a) and liver (fig 2b) of mice that were subjected to low dose endotoxin priming. Contrarily, hardly any thrombi formation is observed in mice not subjected to low dose endotoxin prior to the LPS challenge (difference 24 h low dose priming compared to non-primed mice 6 h after LPS challenge for liver and lung; p < .001). Interestingly, some micro thrombi are already formed after 24 h of low endotoxin priming, before LPS challenge was infused. Apart from isolated inflammatory foci, HE-stained histological sections from kidneys seemed normal in mice subjected to the Shwartzman reaction or a single-dose LPS challenge (not shown).

### (Ischemia-induced) organ damage

**Figure 3:**
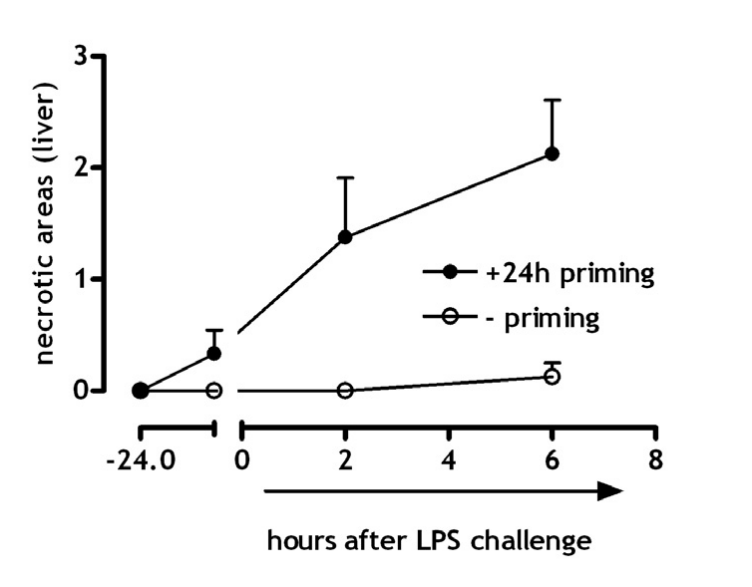
**Hepatic (ischemic) necrosis**. Necrotic areas in the liver were scored for 10 microscopic fields (25×) using haematoxylin and eosin stained sections (mean ± SE)

**Figure 4:**
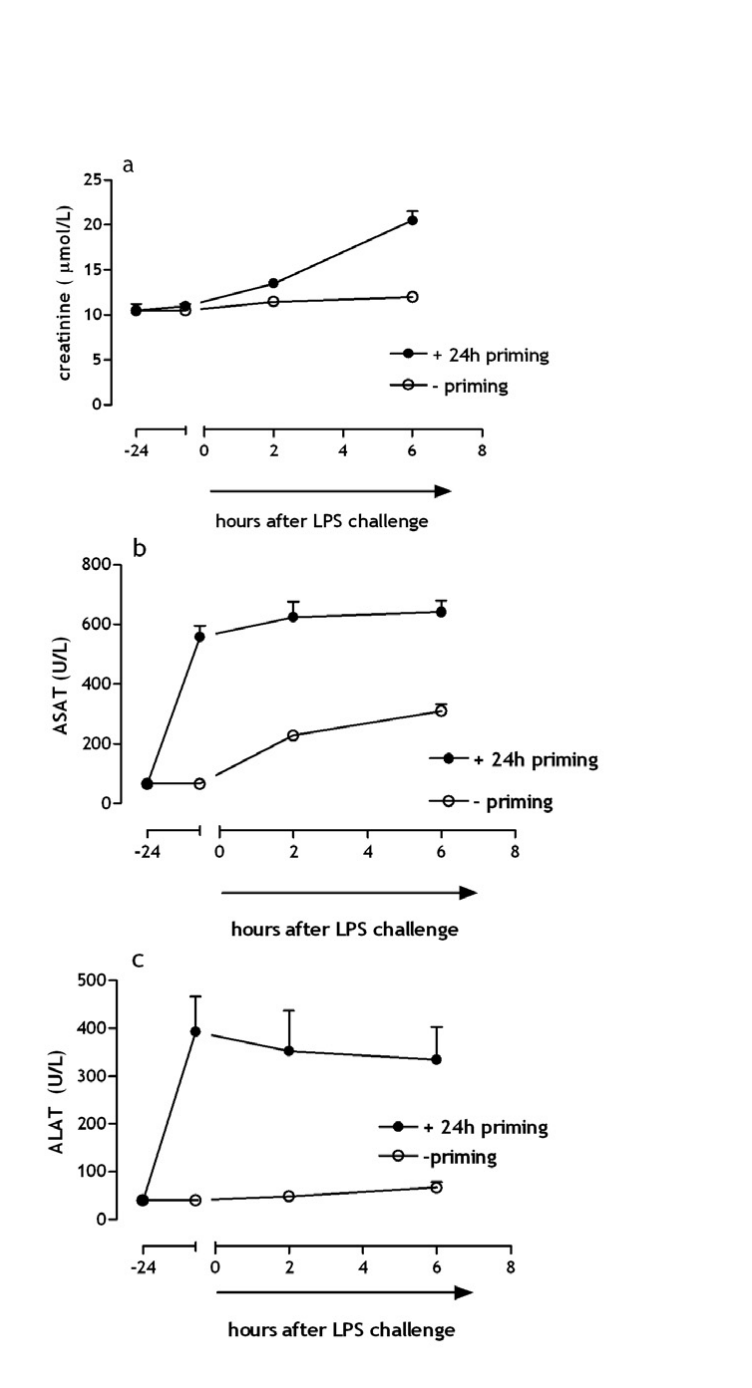
**LPS-induced kidney and liver damage**. (a) Creatinine levels in plasma of mice representing kidney failure. (b+c) Transaminase leakage into plasma reflects acute cellular and mitochondrial hepatic injury (mean ± SE)

Histological examination furthermore displayed significant hepatic necrosis in mice subjected to low dose endotoxin priming; contrarily, necrotic area's in livers of non-primed mice were absent (figure 3; p < .001, 6 h after LPS challenge). In addition to these microscopic differences we also observed macroscopic variability between primed and non-primed mice. Livers of mice exposed to 24 hours of low dose endotoxin priming preceding the LPS challenge exposed a differential degree of ischemic necrosis on the liver surface (not shown). These spots were absent on the surface of the livers of non-primed mice. Figure 3 demonstrates that low dose endotoxin priming prior to an LPS challenge results in ischemic necrosis. Besides coagulation abnormalities and ischemia induced necrosis, DIC may be complicated by (multi-) organ failure and death. Detection of organ damage markers in plasma of mice challenged with LPS demonstrates that low endotoxin priming is critical in the development of both kidney and liver failure. Figure 4a demonstrates that creatinine, a marker for renal failure, is significantly (p < .05) increased 6 hours after LPS challenge, in mice that were previously subjected to low dose endotoxin priming compared to non-primed mice. Creatinine levels in non-primed mice contrarily where not elevated in response to LPS challenge.

In addition, transaminases ASAT and ALAT were highly increased due to the endotoxin priming (fig 4b+c). LPS challenge in non-primed mice also results in a significant increase of the transaminases 6 hours after LPS challenge compared to baseline (fig 4b, ASAT p < .001: fig 3c, ALAT p < 05 ;), the effect, however, was obviously smaller compared to mice that we exposed to the endotoxin priming. These results indicate that low endotoxin priming makes mice more susceptible to a subsequent LPS challenge, resulting in aggravated kidney and liver damage.

## Discussion

The clinical response of sepsis to a systemic inflammatory infection may be complicated by DIC. The syndrome of DIC increases mortality rate and patients with DIC may even respond differently to therapeutic treatment compared to septic patients not hindered by DIC [6]. In order to experimentally study the syndrome of DIC, we aimed for a severe sepsis model complicated by DIC. The Shwartzman reaction has been described as a two-hit animal-model representing DIC [13]. However, most experimental models describing coagulation abnormalities in mice as a consequence of sepsis are based on single dose endotoxemia or live E. coli models. Sustained endotoxin infusions are also regularly used as models for DIC, though predominantly in larger animals like rats and rabbits [19,20].

The initial priming reaction in the Shwartzman phenomenon is a requisite in order to induce lethality [14,21]. In this study we reveal that low-dose endotoxin priming prior to an LPS challenge is accountable for micro-vascular thrombosis and subsequent (multi-) organ failure, which suggests an important role for intravascular thrombi formation contributing to lethality.

Low-dose endotoxin priming, in this study furthermore prominently induced the leak of transaminase enzymes, without further aggravation by LPS challenge. Transaminase leakage is a characteristic for liver dysfunction and reflects acute cellular and mitochondrial injury induced by endotoxin [4,22]. Clinically, secondary liver dysfunction accounts for the spill-over of bacterial and inflammatory mediators, -frequently- leading to multi-organ failure and death [4] In line, LPS challenge in our experimental DIC-model results in augmented hepatic thrombi formation and subsequent necrosis i.e. liver damage.

The aggravation of the LPS response in the Shwartzman reaction is distinctive to the endotoxin tolerance phenomenon wherein low dose endotoxin exposure confers protection against LPS-induced lethality. Endotoxin tolerance is characterized by a markedly reduced immune response and may complicate the management of critically ill patients, particularly patients with severe sepsis [23]. Two phases of endotoxin tolerance have been described: an early phase associated with augmented cellular activation and a late phase associated with the development of specific endotoxin- antibodies. Timing and dosage of low dose endotoxin is critical in endotoxin tolerance [23] as well as the Shwartzman phenomenon [24], apparently resulting in divergent responses. In general, endotoxin tolerance may be considered a state of a dysregulated immunity decreasing the ability to fight infection. The Shwartzman phenomenon on the contrary, affects the coagulatory response aggravating organ dysfunction with minor effects on innate immune responses.

The time interval and dosage of the priming injection are crucial determinants for the Shwartzman reaction to occur [24], the reaction may however also be elicited by two intravenous injections as it will result in similar tissue damage [24]. A local intra-dermal injection is however, easier to manipulate and herein we therefore subjected the mice to an intra-dermal preparatory injection.

Based on the results obtained from this study we conclude that the Shwartzman reaction is better suited to study sepsis-induced DIC adversities compared to a single intravenous endotoxin challenge. In non-primed mice no distinct coagulation abnormalities and/or additional organ dysfunction were observed, suggesting that the (intravenous) single-dose endotoxin challenge is well-suited to study the innate inflammatory response not complicated by DIC.

It should be noted that both endotoxemia models as well as the Shwartzman reaction are obviously different from the clinical situation where a variety of complications occur, these experimental models are thus tentative as a tool to assess new treatment strategies. Endotoxin-induced models may however, be considered appropriate tools to elucidate the underlying pathological mechanisms contributing to sepsis and clotting-related organ failure.

## Conclusion

In this study we demonstrate that low-dose endotoxin priming prior to an LPS challenge in the Shwartzman reaction is accountable for micro-vascular thrombosis and subsequent (multi-) organ failure, not observed in a (intravenous) single-dose endotoxin challenge, which indicates that the Shwartzman reaction is well suited-model to study sepsis-induced DIC adversities.

## Abbreviations

APC; activated protein C

ALAT; Alanine Aminotransferase

ASAT: Aspartate Aminotransferase

ATIII; antithrombin

DIC; disseminated intravascular coagulation

IL-6; interleukin-6

IL1β; interleukin-1β

KC; neutrophil chemotactic protein

LPS; lipopolysaccharide

TFPI; tissue factor pathway inhibitor

TNFα; tumor necrosis factor α

## Competing interests

The author(s) declare that they have no competing interests.

## Authors' contributions

Sjoukje H. Slofstra was involved in planning and performing the study, the analysis of the samples and writing of the manuscript, Hugo ten Cate was involved in the analysis, experimental setup, and writing of the manuscript, C. Arnold Spek was involved in planning, experimental setup, and writing of the manuscript.
